# Supplementary material for: Amphiphilic Graft Copolymers as Templates for the Generation of Binary Metal Oxide Mesoporous Interfacial Layers for Solid-State Photovoltaic Cells
Source: Nanomaterials (Basel). 2024 Feb 13;14(4):352. doi: 10.3390/nano14040352 (PMC10891625; doi:10.3390/nano14040352)
Supplement: Supplementary file 1 [file nanomaterials-14-00352-s001.zip › nanomaterials-2853612-supplementary.pdf]

Supplementary Materials

Amphiphilic Graft Copolymers as Templates for the Generation of Binary Metal Oxide Mesoporous Interfacial Layers for Solid-State Photovoltaic Cells

Seung Man Lim [1], Hayeon Jeong [1], Juyoung Moon [2] and Jung Tae Park [1,*]

1 Department of Chemical Engineering, Konkuk University, 120 Neungdong-ro, Gwangjin-gu, Seoul 05029, Republic of Korea
2 Department of Chemical and Biomolecular Engineering, Yonsei University, 50 Yonsei-ro, Seodaemun-gu, Seoul 03722, Republic of Korea
* Correspondence: : jtpark25@konkuk.ac.kr Tel: +82-2-450-3538, Fax: +82-2-450-8895

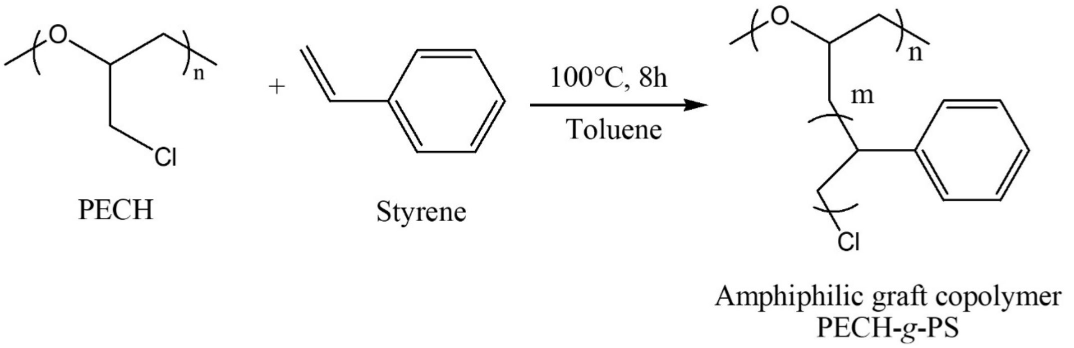

Figure S1. Synthesis of amphiphilic PECH-*g*-PS graft copolymers *via* ATRP.

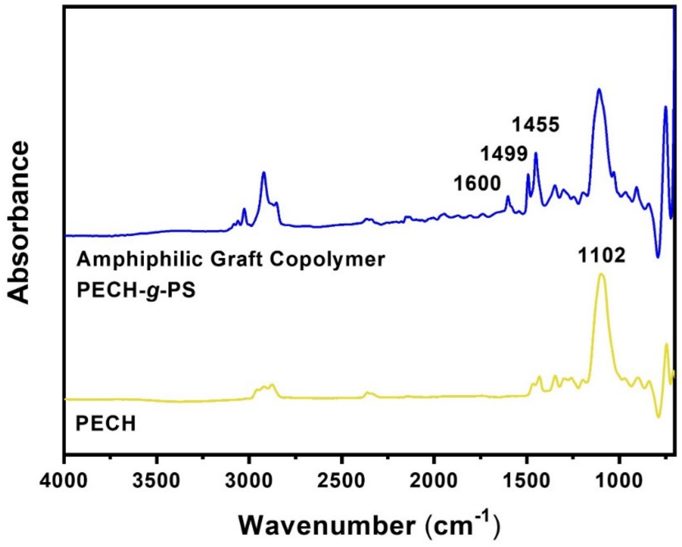

Figure S2. FT-IR spectra of amphiphilic PECH-*g*-PS graft copolymers.

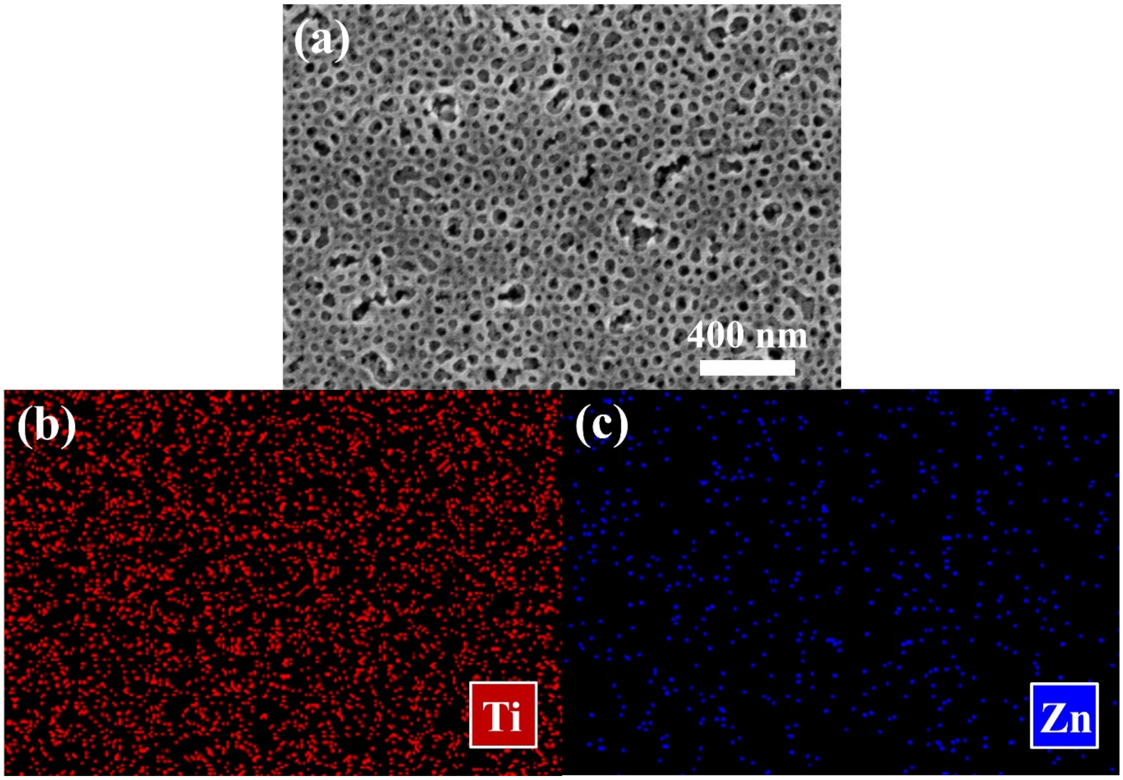

Figure S3. (**a**) SEM-EDS element mapping scan area, (**b**) Ti, and (**c**) Zn elemental maps of bi-MO meso IF layer TZ1.

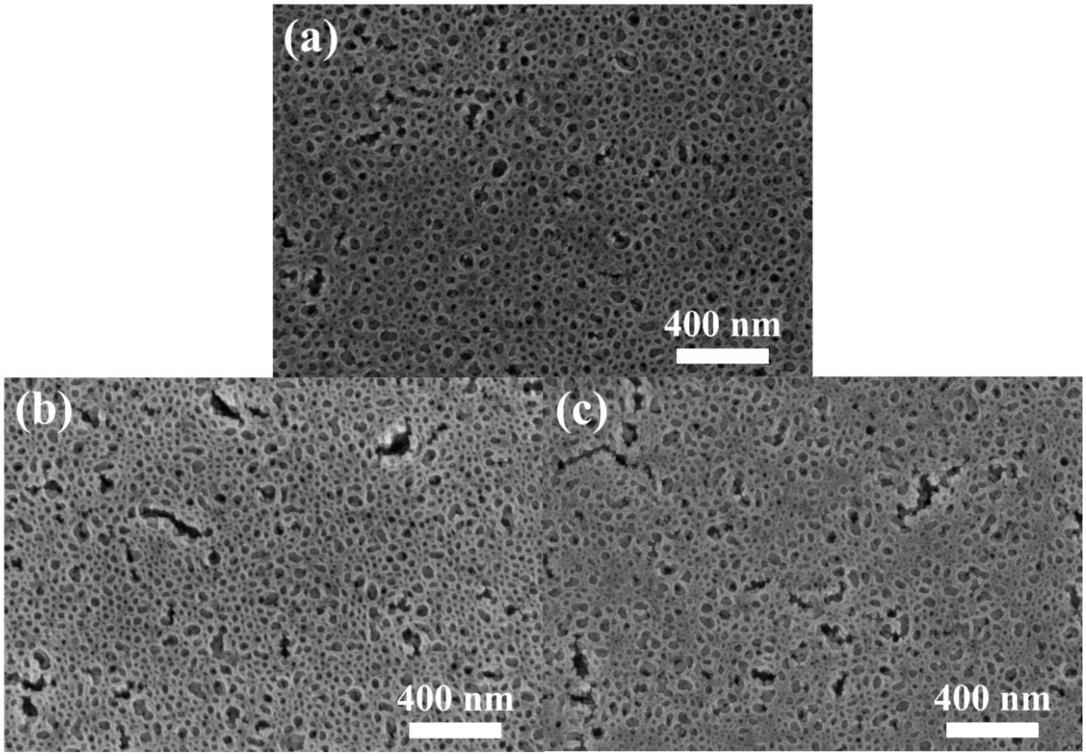

Figure S4. FE-SEM surface images of (**a**) bi-MO meso IF layer TZ0, (**b**) bi-MO meso IF layer TZ2 and (**c**) bi-MO meso IF layer TZ3.

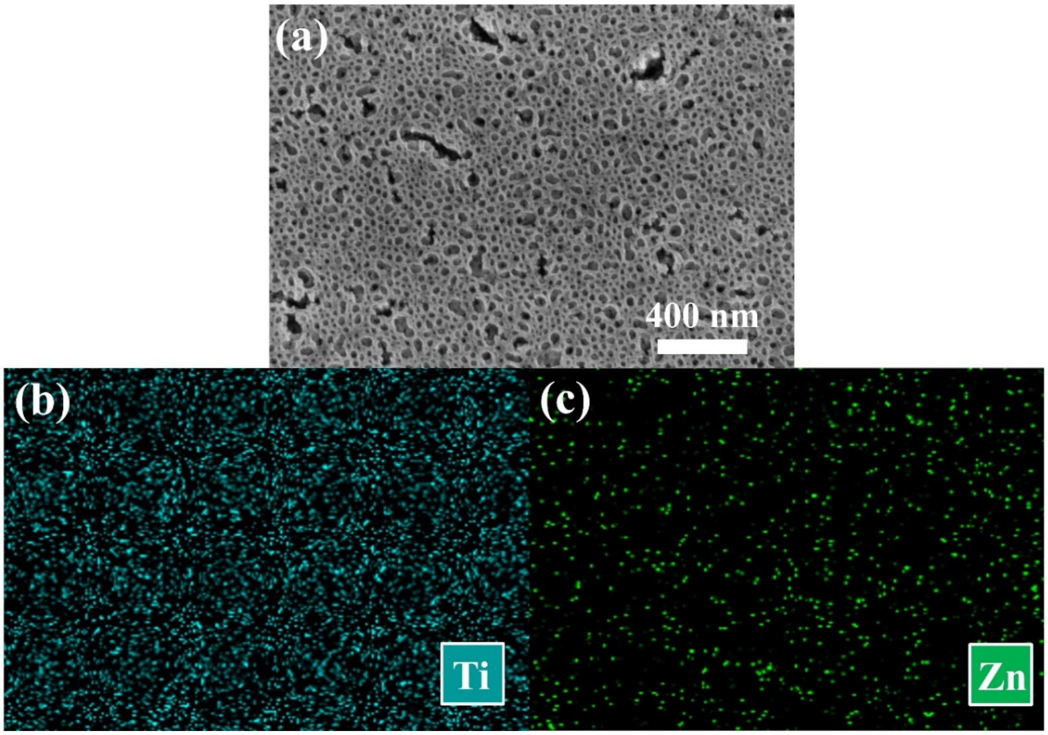

Figure S5. (**a**) SEM-EDS element mapping scan area, (**b**) Ti, and (**c**) Zn elemental maps of bi-MO meso IF layer TZ2.

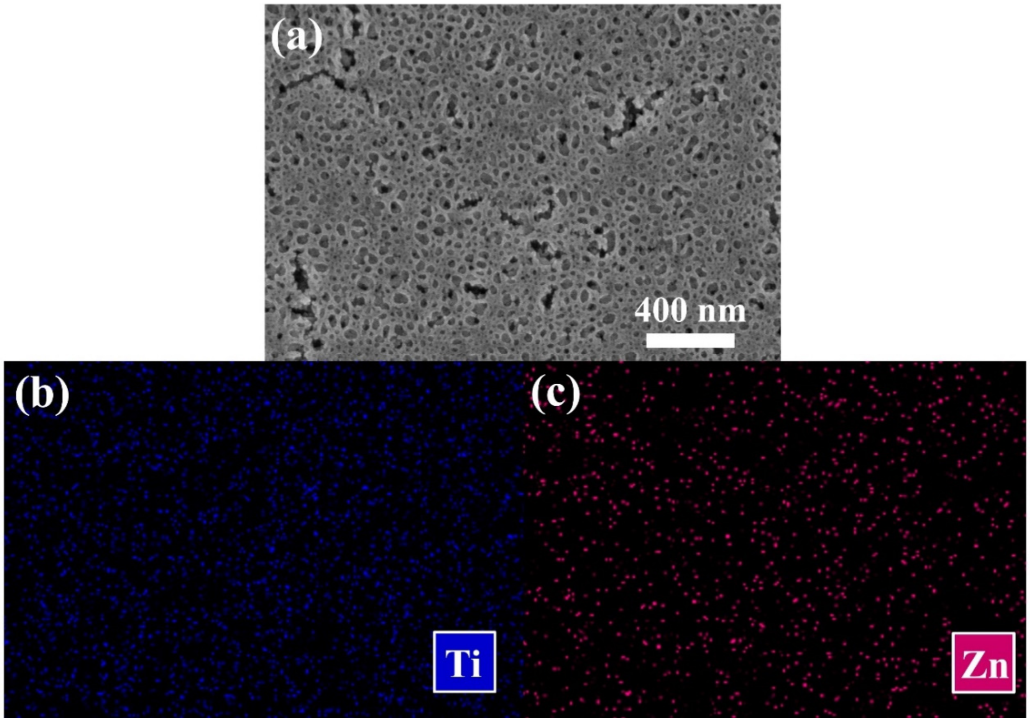

Figure S6. (**a**) SEM-EDS element mapping scan area, (**b**) Ti, and (**c**) Zn elemental maps of bi-MO meso IF layer TZ3.

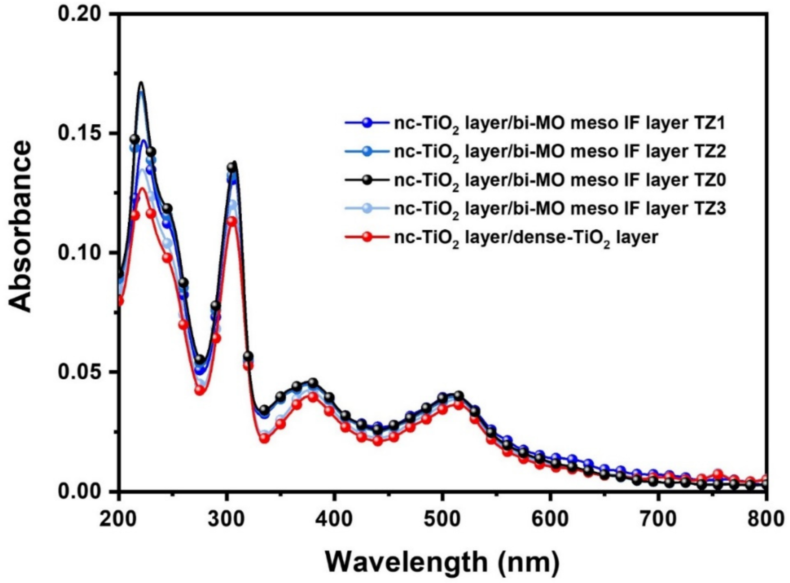

Figure S7. UV-visible spectra of N719 dye loading on bi-MO meso IF layer based solid state photovoltaic cells.

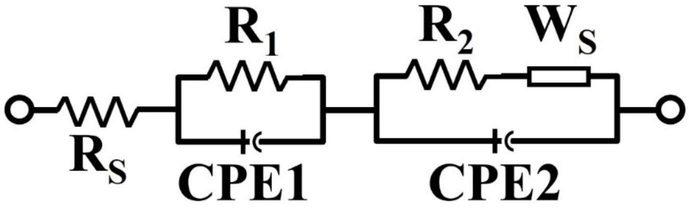

Figure S8. equivalent circuit diagram.

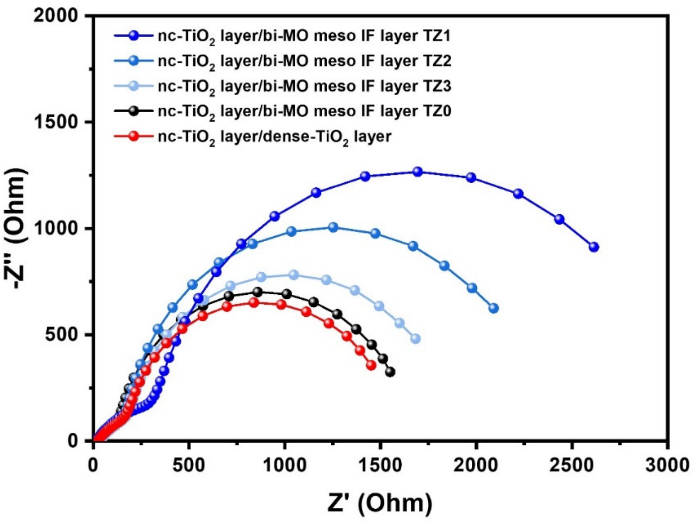

Figure S9. EIS Nyquist plots under dark condition.

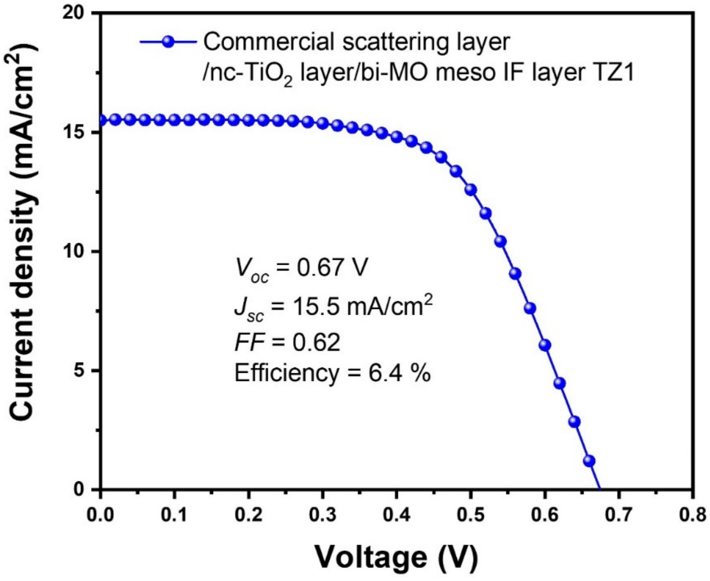

Figure S10. *J–V* curves of bi-MO meso IF layer based solid state photovoltaic cells with commercial scattering layer under 1 sun illumination (AM 1.5 G, 100 mW/cm^2).[a,b,c,d.]

[a] Solid-state electrolyte consists of PEG, LiI, MPII, and I_2 in acetonitrile.
[b] The photoactive area for solid state photovoltaic cells was 0.16 cm^2.
[c] The thickness of the photoanode was approximately 7 μm.
[d] The commercial scattering layer was approximately 5 μm thick.

Table S1. Comparison of photovoltaic parameters of solid state photovoltaic cells with mesoporous layer reported in the literature.

Mesoporous Layer	Solid-State Electrolyte	*Voc* (V)	*Jsc* (mA/cm^2)	*FF*	*η* (%)	Reference
bi-MO meso IF layer	PEG	0.74	9.8	0.69	5.0	This work
bi-MO meso IF layer w/ Scattering layer	PEG	0.67	15.5	0.62	6.4	This work
Mesoporous TiO_2 bead	HPC	0.70	16.5	0.67	7.8	[48]
TiO_2 film	MXene	0.78	15.0	0.66	7.8	[49]
TiO_2 film	Cs_2SnI_6	0.62	10.62	0.62	4.1	[50]
TiO_2 film	PEG/WO_3	0.71	14.6	0.61	6.3	[51]
TiO_2 film	PEG/ZrO_2	0.66	14.0	0.61	5.6	[52]
TiO_2 film	PEG	0.64	13.6	0.65	5.7	[53]
TiO_2 film	PEG/ZIF-67	0.68	10.1	0.65	4.6	[54]
